# INFORMAL DEMENTIA CAREGIVERS' EMOTIONAL EXPERIENCES DURING CONFLICT AND DEPRESSIVE SYMPTOMS

**DOI:** 10.1093/geroni/igac059.1576

**Published:** 2022-12-20

**Authors:** Casey Brown, Yuxuan Chen, Robert Levenson

**Affiliations:** Georgetown University, Washington, District of Columbia, United States; UC Berkeley, Berkeley, California, United States; University of California, Berkeley, Berkeley, California, United States

## Abstract

Emotional interactions may change as a result of dementia and negatively impact caregivers’ mental health. We examined 62 dementia caregivers’ emotional experiences during conflict with their partners, including 22 individuals with behavioral variant frontotemporal dementia (bvFTD), 20 with Alzheimer’s disease (AD), and 20 with a language variant of frontotemporal dementia (language variant). Dyads engaged in a 10-minute conversation involving conflict. Caregivers watched recordings of their conversation while rating their emotional valence using a rating dial. Caregivers of individuals with bvFTD reported greater decreases in positive emotion across the conversation relative to caregivers of individuals with AD and language variants. Caregivers who reported greater decreases in positive emotion across the conversation had higher levels of depressive symptoms, even after accounting for their partner’s diagnosis and level of cognitive impairment. Findings suggest bvFTD caregivers experience the greatest declines in positive emotion during conflict, with potential implications for depression.

